# Cross-sectional analysis reveals COVID-19 pandemic community lockdown was linked to dysregulated cortisol and salivary alpha amylase in children

**DOI:** 10.3389/fpubh.2023.1210122

**Published:** 2023-12-15

**Authors:** Katherine M. Lloyd, Laurel Gabard-Durnam, Kayleigh Beaudry, Michael De Lisio, Lauren B. Raine, Ysabeau Bernard-Willis, Jennifer N. H. Watrous, Susan Whitfield-Gabrieli, Arthur F. Kramer, Charles H. Hillman

**Affiliations:** ^1^Department of Psychology, Northeastern University, Boston, MA, United States; ^2^Department of Cellular and Molecular Medicine, University of Ottawa, Ottawa, ON, Canada; ^3^Department of Physical Therapy, Movement, and Rehabilitation Sciences, Northeastern University, Boston, MA, United States; ^4^Division of Cognitive and Behavioral Neurology at Brigham and Women’s Hospital, Boston, MA, United States; ^5^Department of Psychology, Oklahoma State University, Tulsa, OK, United States; ^6^University of Illinois Beckman Institute, Champaign-Urbana, IL, United States

**Keywords:** children, cortisol, salivary alpha amylase, stress, dysregulation, COVID- 19

## Abstract

The COVID-19 pandemic altered everyday life starting in March 2020. These alterations extended to the lives of children as their normal routines were disrupted by community lockdowns, online learning, limited in-person social contact, increased screen time, and reduced physical activity. Considerable research has investigated the physical health impact of COVID-19 infection, but far fewer studies have investigated the physiological impact of stressful pandemic-related changes to daily life, especially in children. The purpose of this study was to leverage an ongoing clinical trial to investigate physiological consequences associated with chronic stress of pandemic community lockdown on children. As a part of the clinical trial, children provided saliva samples. Saliva samples were analyzed for cortisol and salivary alpha amylase (sAA) content. This secondary cross-sectional analysis included 94 preadolescent children located within the Greater Boston, Massachusetts community. Children participated in the study either before, during, or following the pandemic community lockdown to form three groups for comparison. In response to chronic stress caused by the pandemic community lockdown, participants demonstrated dysregulation of fast-acting catecholamine response of the locus-coeruleus-norepinephrine system and slower-acting glucocorticoid response, resulting in an asymmetrical relationship of hypocortisolism (*M* = 0.78 ± 0.19 μg/mL, *p* < 0.001) paired with higher sAA (*M* = 12.73 ± 4.06 U/mL, *p* = 0.01). Results suggest that the abrupt COVID-19 disruption to daily life, including the stressful experience of community lockdown, had physiological effects on typically developing children. Further research is required to investigate mental health outcomes of children following the chronic stress of the pandemic community lockdown.

## Introduction

The World Health Organization declared COVID-19 a pandemic in March 2020, after 110 countries and territories reported cases of the illness (1). In the United States, community lockdowns mandated nonessential activities be cancelled, physical distancing be employed, and travel postponed. This allowed the government to detect, isolate, test, and care for cases, and to trace and quarantine contacts with the infected. However, these drastic measures altered the landscape of individuals’ daily lives, especially for children (2).

On March 13^th^, 2020, the State of Massachusetts suspended in-person schooling. By April 8th, 2020, 188 countries globally suspended in-person schooling (3). While some children may have benefitted from increased parental interaction and less school bullying (4), many children experienced heightened levels of emotional distress (5). Children were socially and physically isolated for months during prolonged school closures without connection to friends, teachers, extended family, or community support (6). Many parents lost their jobs, leading to financial stress in the family (7). Virtual learning was inaccessible for many children with inadequate technology at home (8) and difficult for students with learning disabilities and special needs (9). Less exercise, more screen time, and increased snacking lead to increased body mass index, reduced cardiorespiratory fitness, and poorer sleep quality in children (4, 10–12). In addition, for low-income children, prolonged school closures also resulted in food insecurity without easy access to meals at school (13). Lastly, children experienced COVID-related illness in their families and grieved deaths (14). These stressors compounded over time and resulted in a long-term chronic stress event for many people, especially children.

We are only just beginning to understand the long-term psychological effects of community lockdown. While children are resilient to adversity, they are not immune to it (15). Childhood is a vulnerable window of cognitive development, during which sustained and augmented stressors, particularly social isolation, can affect mental health long-term (16–18). For example, a study in 2020 evaluated 1,036 quarantined children and adolescents in China ages 6–15 years old, and found that 11.78% of children had depression, 18.92% had anxiety, and 6.56% presented with both depression and anxiety (19). A study in India revealed that 66.11% of their sample of 121 quarantined children experienced helplessness, 68.59% worry, and 61.98% fear (20). In addition, several key meta-analyses and systematic reviews have concluded that children and adolescents showed increased depression, anxiety, sleep disorders, post-traumatic stress symptoms, poor appetite, inattentiveness, and significant separation anxiety as a result of the pandemic and community lockdown measures (4, 21–24). Most severely, the mental health crisis stemming from the pandemic and community lockdown measures has resulted in increased suicide (25), suicidal ideation (26), and a 50.6% increase in mean emergency department visits for suspected suicide attempts among girls aged 12–17 years old (27). Most large reviews on the psychological and behavioral outcomes of children during the community lockdown use self-reported data, and we have yet to link a mechanistic cause to the observed pandemic-related changes in mental health.

One such mechanistic cause may be physiological changes in response to chronic stress. The typical stress response involves a fast-acting catecholamine response by activation of the locus coeruleus-norepinephrine (LC-NE) system. The LC-NE system controls arousal, alertness, and vigilance by supplying norepinephrine to the amygdala, hippocampus, prefrontal cortex, and cerebellum (28–30). LC-NE activity can be quantified using a salivary biomarker called alpha amylase produced by acinar cells which are innervated by the sympathetic nervous system (31). In addition to the fast-acting catecholamine response, the hypothalamic–pituitary–adrenal (HPA) axis activates a slower-acting glucocorticoid response returns the body to a state of physiological equilibrium (32). HPA activity can be quantified by analyzing the hormone cortisol in saliva. Although unique, the LC-NE system and HPA axis share rich intercommunication, as they are structurally and functionally related (33). Similarly, during typical stress response, salivary alpha amylase (sAA) and cortisol are correlated with one another when the lag time following the onset of the stressor is accounted for. However, during a period of severe, chronic stress, these responses can become dysregulated and the coordination between the pathways can deteriorate (33, 34), resulting in atypical stress response. For example, atypical stress response may result in hypocortisolism, which is unexpectedly low cortisol and blunted cortisol release. In adults, hypocortisolism has been linked to feelings of depression, apathy, irritability, difficulty concentrating, confusion, stress sensitivity, and poorer memory that can begin in childhood and continue into adulthood (35). In extreme cases of traumatic early life experiences, higher sAA and hypocortisolism have been observed in tandem in individuals, further highlighting dysregulation and deterioration in communication between pathways in response to chronic stress (36). Therefore, both the faster and slower-acting stress responses are important to include in analysis for a complete picture into typical and atypical stress response.

Given the chronic stress associated with the COVID-19 pandemic, the isolation related to community lockdowns, and observed decrease in mental health and behavior in children, we aimed to investigate whether the chronic stress of the pandemic community lockdown affected physiological outcomes in preadolescent children. We hypothesized that the largest observable differences in physiological changes would be found for cortisol compared to sAA, as the HPA-axis responds more slowly to stressors and would better represent typical stress for participants. We hypothesized that the slower-acting glucocorticoid response would be dysregulated during the isolation and extreme chronic stress of the pandemic community lockdown, manifesting as hypocortisolism in children. We also hypothesized that we would observe an asymmetrical stress response with higher sAA coupled with lower cortisol during the lockdown, signaling a breakdown in the communication between the LC-NE and HPA mechanistic pathways. By understanding the physiological changes associated with the chronic stress of the pandemic community lockdown, we can begin to understand a potential mechanistic cause of COVID-19 pandemic-related changes in mental health, and prompt social and mental support for children following the community lockdown.

## Materials and methods

### Participants

This cross-sectional investigation was a secondary analysis of a larger clinical trial (ClinicalTrials.gov Identifier: NCT03592238). The inclusion criteria for participants included: parental/guardian consent and participant assent, between the 9–10 years of age, capable of performing exercise, normal/average intelligence quotient (IQ) or above (i.e., >85), classified as pre-pubescent or in the earliest stages of puberty (37), no prior diagnosis of cognitive or physical disability, not taking any anti-psychotic, anti-depressant, anti-anxiety, or attention deficit disorder (ADD)/ attention deficit hyperactivity disorder (ADHD) medications, normal or corrected-to-normal vision, and able to speak and read English (38). The detailed methodology is provided in a recent publication (38). In brief, healthy, typically developing children from the Greater Boston, Massachusetts community were included in the study. One hundred and three participants provided written informed assent and their legal guardians provided written informed consent in accordance with the Northeastern University Institutional Review Board, the University of Ottawa Research Ethics Board, and the Declaration of Helsinki for human studies. Ninety-four participants were included in analysis (38% female), and 9 participants were not included in analysis due to unique, extenuating circumstances that resulted in long-term disruptions to their study timeline (i.e., started participation in before the community lockdown and ended following the community lockdown). Table 1 provides the demographic information for all participants included in analysis.

**Table 1:** Participant demographics.

	Before lockdown (*n* = 40)	During lockdown (*n* = 25)	After lockdown (*n* = 29)
Age (years)	9.91 ± 0.56	10.40 ± 0.64	10.10 ± 0.59
Sex (%Female)	42.5%	32.0%	37.9%
Race	60% White or Caucasian 10% African American 7.5% Asian 22.5% Mixed or other	72% White or Caucasian 8% African American 7.5% Asian 16% Mixed or other	51.7% White or Caucasian 13.8% African American 13.8% Asian 17.2% Mixed or other
Ethnicity (%Hispanic)	15%	4%	10.3%
Pubertal status	1.36 ± 0.57	1.72 ± 0.76	1.38 ± 0.48
Mother’s education	2.5% High school graduate 12.5% Some college 40% Bachelor’s degree 45% Advanced degree	0% High school graduate 16% Some college 20% Bachelor’s degree 60% Advanced degree 4% abstained from answering	0% High school graduate 3.4% Some college 31% Bachelor’s degree 62.1% Advanced degree 3.4% abstained from answering
Household income	2.5% $21,000–$30,000 2.5% $31,000–$40,000 5.0% $41,000–$50,000 5.0% $51,000–$60,000 10.0% $61,000–$70,000 5.0% $71,000–$80,000 2.5% $91,000–$100,000 67.5% > $100,000	4.0% $21,000–$30,000 4.0% $31,000–$40,000 4.0% $41,000–$50,000 4.0% $51,000–$60,000 8.0% $61,000–$70,000 12.0% $71,000–$80,000 4.0% $91,000–$100,000 60.0% > $100,000	3.4% $5,000-11,999 3.4% $50,000-74,999 6.9% $75,000–$99,000 65.5% $100,000–$199,999 13.8% > $200,000 3.4% abstained from answering

The values reflect mean ± SD. An updated scale was used for household income for After Lockdown participants, and thus, statistical differences between groups could not be observed.

### Procedure

Children practiced passive saliva collection on their screening visit to ensure that they could provide adequate sample volume (≥ 2 mL). Participants were not instructed to fast for the screening visit, so the practice sample saliva collection was discarded and not analyzed. In this study, unstimulated salivary sampling was used. Once seated comfortably, the participant leaned forward, put their elbows on their knees, tucked their chin to their chest to put pressure on their salivary glands, and collected saliva in their mouth for 2 min. At the end of the 2 min, the child raised a funneled collection tube to their lips and used their tongue to gently guide the saliva into the tube.

**Figure 1:**
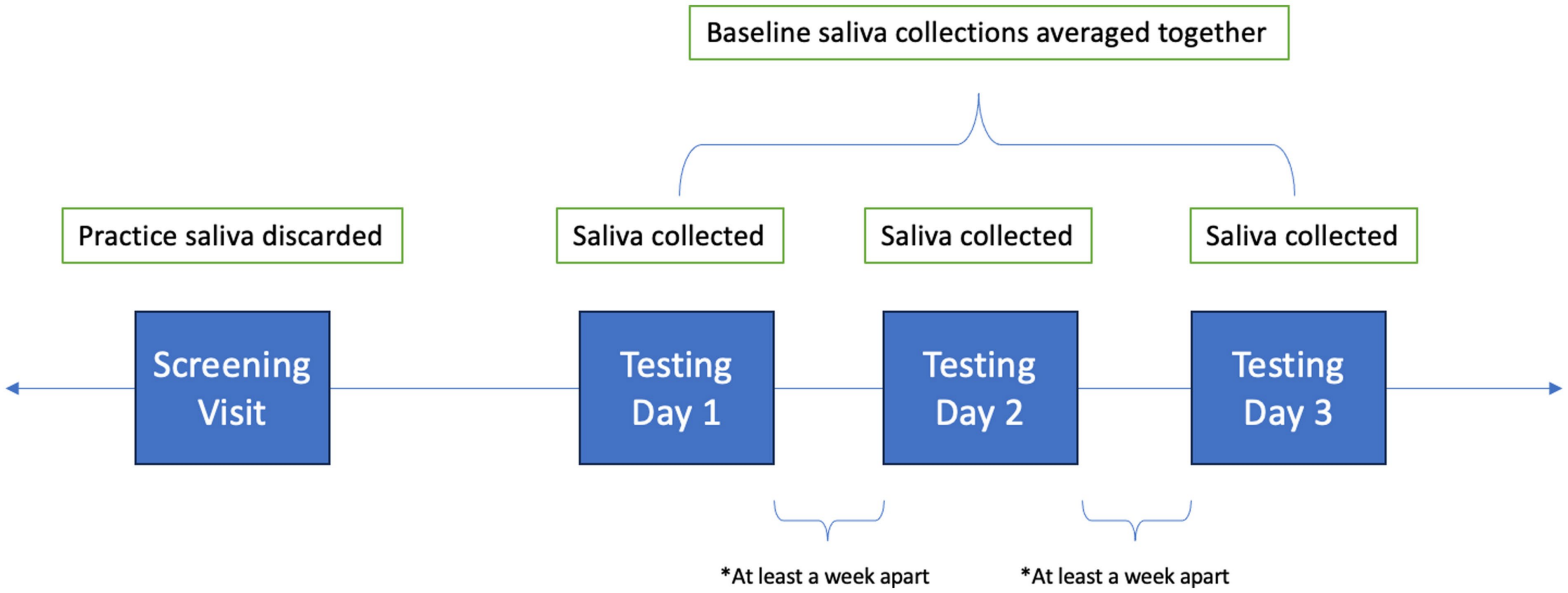
Timeline of Saliva Collections.

Following the screening visit, participants returned to the lab for three more testing days (see Figure 1). Testing days were scheduled at least a week apart at the same time of day to account for diurnal variation. Testing days were also scheduled for when children did not have structured physical education or sports planned prior to testing. Children provided 4 saliva samples throughout each testing day, but only the first collection was used for analysis, as it was collected prior to testing procedures. All saliva collections occurred in the lab, and all collections had to fall into the predetermined time frames of before, during, or after the community lockdown. Children were asked not to consume any food or beverages besides water for at least 1 h prior to visiting the laboratory. Further, they were asked to discontinue consumption of foods high in caffeine, sugar, or acidity 6 h prior to arrival, and not to brush their teeth less than 1 h prior to arrival. Participants were also asked to report any canker sores, oral diseases, oral injuries, or medication consumption within the last 12 h. Collection tubes were weighed before and after collection with the cap on. Samples were aliquoted and stored in −80°C freezer until analysis.

sAA and cortisol samples were shipped on dry ice and were analyzed at University of Ottawa using *α*-amylase Saliva Enzymatic Assay and Cortisol Saliva ELISA (Hamburg, Germany). Upon arrival at the University of Ottawa, samples were thawed, centrifuged at 3000 x *g* for 15 min at room temperature. sAA was analyzed according to manufacturer’s instructions (IBL International, Hamburg, Germany, RE80111). Briefly, diluted samples (1:301) were mixed with substrate solution in duplicate. Readings were conducted using a microplate reader (POLARstar Omega, BMG Labtech, Guelph, Canada) at 22°C at a wavelength of 405 nm at 3 and 8 min after incubation at room temperature. Cortisol was analyzed according to manufacturer’s instructions (IBL International, Hamburg, Germany, RE852611). Briefly, duplicate samples were incubated with enzyme conjugate solution at room temperature for 2 h, washed, and incubated with enzyme substrate solution at room temperature for 30 min. “Stop Solution” was applied to end the enzyme/substrate reaction, and the samples were read in a microplate reader (POLARstar Omega, BMG Labtech, Guelph, Canada) at 450 nm within 15 min. Sample results were averaged across three testing days. All data were normalized to standard curves prior to statistical analysis. To reduce positive skew, cortisol data were subject to log transformation and sAA data were subject to square root transformation (39, 40).

### Statistical analysis

Analyses were conducted using SPSS (v25) and RStudio (v2021.09.0) with *p* = 0.05. Children were compared by lockdown status: Before (*n* = 40), During (*n* = 25), and After (*n* = 29) Lockdown. We defined lockdown start date as March 13th, 2020 (when schools closed in Massachusetts), and end date as April 26th, 2021 (when in-person schooling resumed). Chi-square tests of independence and ANOVAs were performed to determine if groups differed between age, race, sex, ethnicity, pubertal status, or mother’s education. A *t*-test was performed to determine if more males were included in analysis compared to females.

To ensure transformed data passed the normality assumption, a Shapiro–Wilk test was performed for sAA and cortisol. For salivary outcomes, age, sex, and time of day the samples were used as covariates in analyses due to their known influence on sAA and cortisol (41, 42). ANCOVAs were performed to determine if sAA or cortisol differed based on lockdown status. Post-hoc t-tests with Bonferroni correction for multiple comparisons were conducted to determine significant contrasts. To provide additional context to the salivary outcomes, additional ANCOVAs and correlations were performed to determine if physiological changes in stress response were related to demographic variables.

## Results

Table 1 describes participant demographics of the sample. The groups based on lockdown status did not differ based on race [*X*^2^ (8, *N* = 94) = 5.62, *p* = 0.69], sex [*X*^2^ (2, *N* = 94) = 0.72, *p* = 0.70], ethnicity [*X*^2^ (4, *N* = 94) = 4.47, *p* = 0.35], pubertal status [*F* (2, 86) = 2.88, *p* = 0.06, *η*^2^ = 0.06], or mother’s education [*X*^2^ (8, *N* = 94) = 8.11, *p* = 0.42]. Cortisol did not differ based on race [*F* (4, 85) = 2.19, *p* = 0.08, *η*^2^ = 0.09], ethnicity [F (4, 85) = 2.19, *p* = 0.08, *η*^2^ = 0.09], or mother’s education [*F* (4, 86) = 1.52, *p* = 0.21, *η*^2^ = 0.07]. Similarly, sAA did not differ based on race [F (4, 86) = 0.89, *p* = 0.47, *η*^2^ = 0.04], ethnicity [*F* (2, 88) = 0.35, *p* = 0.71, *η*^2^ = 0.01], or mother’s education [*F* (4, 86) = 0.23, *p* = 0.92, *η*^2^ = 0.01]. In this study, more males (*n* = 58) were included in analysis compared to females (*n* = 36) [*t* (93) = 27.44, *p* < 0.001, *d* = 2.83, 95% CI (2.37, 3.28)]. Neither sAA [t (92) = 0.57, *p* = 0.57, *d* = 0.12, 95% CI (−0.54, 0.30)] nor cortisol [*t* (92) = 1.00, *p* = 0.32, *d* = 0.21, 95% CI (−0.63, 0.21)] significantly differed between males and females in the sample. Age significantly differed by lockdown status [*F* (2, 90) = 5.45, *p* = 0.01, *η*^2^ = 0.11, 95% CI (0.01, 0.23)]. Post-hoc comparisons revealed significantly lower age in the before lockdown group (*M =* 9.91 ± 0.56 years) compared to during lockdown group (*M =* 10.40 ± 0.64 years). Age was not significantly correlated with sAA [*r* (93) = 0.15, *p* = 0.15] or cortisol [*r* (93) = −0.22, *p* = 0.84]. Pubertal status was not significantly correlated with sAA [*r* (89) = 0.09, *p* = 0.41] or cortisol [*r* (89) = −0.02, *p* = 0.83]. Specifically, no significant correlation was observed for male pubertal status for sAA [*r* (55) = 0.14, *p* = 0.32] or cortisol [*r* (55) = −0.15, *p* = 0.26], or female pubertal status for sAA [*r* (34) = 0.01, *p* = 0.97] or cortisol [*r* (34) = 0.05, *p* = 0.78].

**Figure 2:**
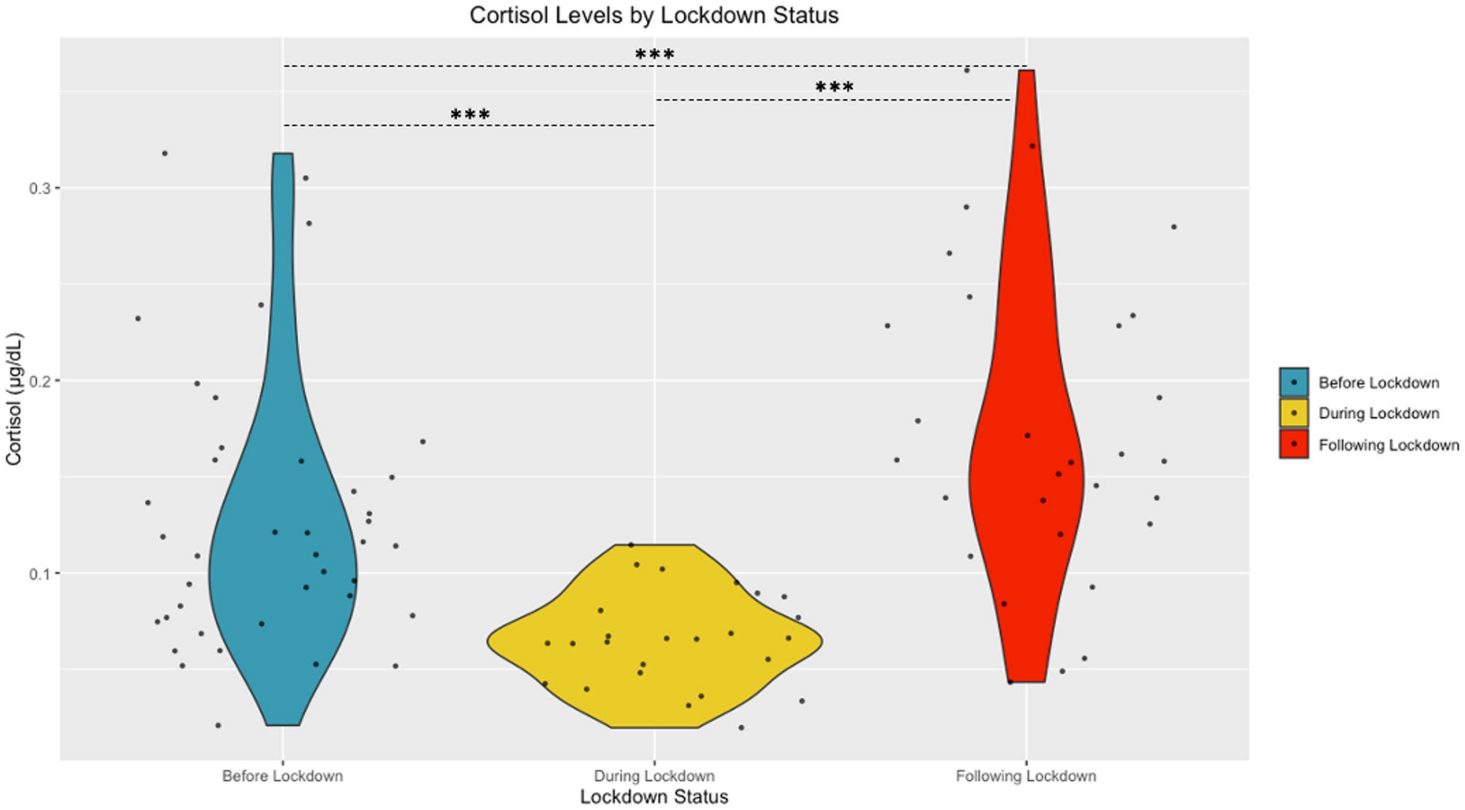
Salivary Cortisol by Lockdown Status (Untransformed Data Shown).

**Figure 3:**
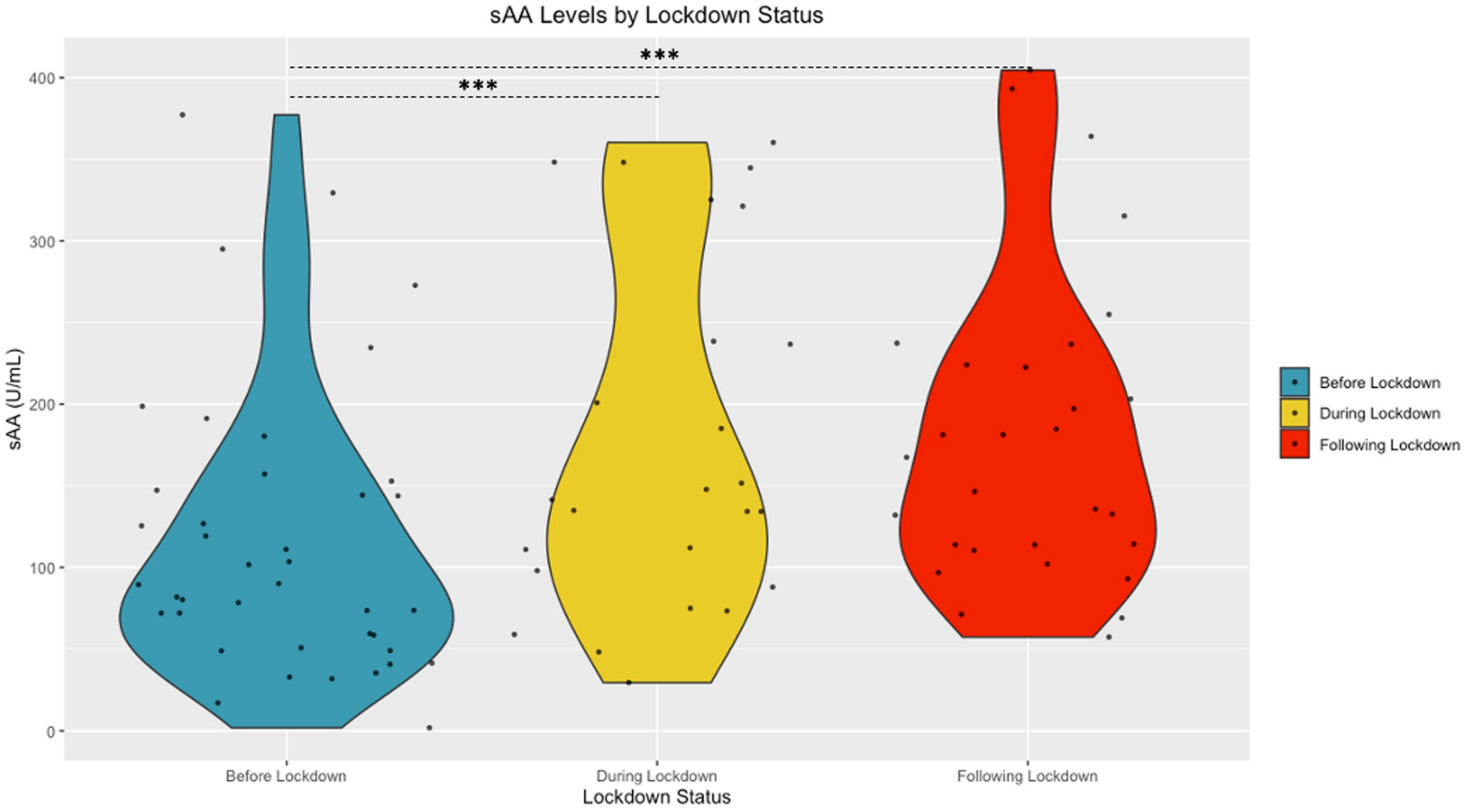
Salivary sAA by Lockdown Status (Untransformed Data Shown).

The Shapiro Wilk tests revealed that sAA [W (94) = 0.98, *p* = 0.15] and cortisol [W (94) = 0.99, *p* = 0.58] had normal distributions following transformation. A significant difference in cortisol based on community lockdown status was observed [*F* (2, 87) = 22.53, *p* < 0.001, *η*^2^ = 0.33] (see Figure 2). Post-hoc comparisons revealed significantly lower cortisol During Lockdown (*M* = 0.78 ± 0.19 μg/mL) compared to Before (*M* = 1.05 ± 0.24 μg/mL) and After (*M* = 1.19 ± 0.23 μg/mL) Lockdown. Before Lockdown (*M* = 1.05 ± 0.24 μg/mL) children also exhibited lower cortisol compared to After Lockdown (*M* = 1.19 ± 0.23 μg/mL). A significant difference in sAA based on lockdown status was also observed [*F* (2, 87) = 4.67, *p* = 0.01, η^2^ = 0.10] (see Figure 3). Post-hoc comparisons revealed significantly higher sAA During Lockdown (*M* = 12.73 ± 4.06 U/mL) compared to Before Lockdown (*M* = 10.12 ± 3.88 U/mL). After Lockdown (*M* = 13.05 ± 3.38 U/mL) children also exhibited higher sAA compared to Before Lockdown (*M* = 10.12 ± 3.88 U/mL).

## Discussion

The main findings from this study suggest that the abrupt COVID-19 disruption to daily life, including the stressful experience of community lockdown, had physiological effects on developing children. We observed that during the pandemic community lockdown, preadolescent children presented with hypocortisolism. Sustained stress over a long period of time or an intensely stressful situation can drive the HPA axis to elevate levels of cortisol continuously, resulting in an adaptation in which less cortisol is released by the hypothalamus and pituitary. Hypocortisolism is meant to be protective to ensure long-term survival by preventing chronically high cortisol levels from suppressing immune function and increasing catabolic pathways (35). The magnitude of the HPA axis response to stressor is determined by (1) the novelty to the individual, (2) unpredictable nature, (3) threat to their person or ego, and (4) sense of loss of control (35). Blunted cortisol response as a result of chronic stress has negative consequences on other physiological systems and can damage mental and physical health. For example, animal studies investigating early-life adversity on the development of the stress response has been well-documented. Rodent pups separated from their mother have shown heightened anxiety, fearful behaviors, and hypervigilance across the rodent lifespan (43–47). Similarly, rhesus (48), marmoset (49), and squirrel monkeys (50) exposed to early-life adversity and isolation have also demonstrated hypocortisolism. In addition to animal research, dysregulated cortisol and sAA have been observed in children with early-life adversity including adoption (51), poverty (52), and sexual abuse (53). Asymmetry of lower cortisol coupled with higher sAA observed during lockdown has also been observed in the context of children experiencing extended marital conflict (36) and maltreatment (54). Critically, physiological changes in response to chronic stress in children has been linked to fatigue, depression, apathy, sleep disturbances, stress sensitivity, difficulty with memory, and other negative health outcomes that can continue into adulthood (35) and potentially result in later-life mental disorders, aggression, substance use, and attention problems (55, 56). Encouragingly, animal and human research has shown that negative outcomes caused by early-life adversity can be partially ameliorated by placement in enriched (57) and supportive (e.g., learning and educational supports, childcare, emotional support, specialized therapy, healthcare, etc.) environments (58).

This study’s limitations are rooted in the unpredictability of the pandemic community lockdown – we did not know a lockdown would occur, how long it would last, or what variables would be important to collect during this time. Should another pandemic community lockdown occur again to prevent the spread of disease, future research should adhere to the following recommendations. A limitation of this study is that due to the community lockdown, a considerable gap in data collection, lasting more than several months, exists in our dataset between when salivary measures and mental health measures were collected. As such, we were unable to assess participants’ salivary measures when the mental health measures were collected. Therefore, for future research, we recommend synchronous collection of salivary and mental health measures to better investigate potential relationships. Extending our promising preliminary cross-sectional results, we recommend longitudinal research with repeated salivary and mental health measures with a control group, if possible. In future research, age must be considered, as human studies have shown that basal sAA and cortisol increase significantly throughout puberty into adolescence (59–61). And, once puberty begins, there are clear sex differences in stress response (62–64). We also recommend recruiting more participants for analysis – we included 94 participants but required 102 to be adequately powered for large effect sizes (*η*^2^ ≥ 0.14). We also recommend collecting information regarding vaccination, contraction of COVID-19, if parents worked from home or were essential workers, if any family members or friends passed away from the disease, and asking children to rate how stressful lockdown was for them and their family.

Our results suggested physiological changes in response to chronic stress, but future research should attempt to tie physiological changes to observed changes in mental health. Research has only just begun to explore the relationship between hypocortisolism, mental health, and the COVID-19 pandemic. Previous COVID-19 research has explored hypocortisolism using hair cortisol samples and observed relationships with child emotional-behavioral health (65). Further, loneliness in children during the COVID-19 pandemic was related to blunted cortisol awakening responses (66). The COVID-19 pandemic-related chronic stress has resulted in long-term impacts on mental health (67), and our results suggest a candidate mechanism, HPA dysregulation, for these changes in mental health.

We aimed to investigate how the chronic stress of the pandemic community locked affected physiological outcomes in preadolescent children. This is the first study to compare salivary biomarkers in children before, during, and following the COVID-19 community lockdown. While this secondary analysis faced data collection limitations, we were clearly able to observe clear dysregulated cortisol and sAA during the community lockdown. This cross-sectional research provides an important window into the lives of preadolescent children during a time of chronic stress. Previous literature has connected similar physiological changes to early life adversity and poorer mental health, prompting the need for future research to connect changes in these biomarkers to changes in mental health. These findings suggest a potential mechanistic cause for the COVID-19 pandemic-related changes in mental health, emphasizing the need for social and mental support for children following the COVID-19 pandemic.

## Data availability statement

The datasets generated and analyzed during the current study are available from the corresponding author on reasonable request, following the publication of the study’s main aims.

## Ethics statement

The studies involving humans were approved by the Northeastern University Institutional Review Board, the University of Ottawa Research Ethics Board, and the Declaration of Helsinki for human studies. The studies were conducted in accordance with the local legislation and institutional requirements. Written informed consent for participation in this study was provided by the participants’ legal guardians/next of kin.

## Author contributions

All authors listed have made a substantial, direct, and intellectual contribution to the work and approved it for publication.
